# Investigating the Role of DNA Ploidy and Proliferation Index in Distinguishing Ameloblastoma from Ameloblastic Carcinoma

**DOI:** 10.1007/s12105-024-01742-2

**Published:** 2025-01-30

**Authors:** Liam Robinson, Chané Smit, Marlene B. van Heerden, Melvin A. Ambele, Willie F. P. van Heerden

**Affiliations:** 1 Department of Oral and Maxillofacial Pathology, Faculty of Health Sciences, University of Pretoria, Pretoria, South Africa; 2 Institute for Cellular and Molecular Medicine, Extramural Unit for Stem Cell Research and Therapy, Faculty of Health Sciences, South African Medical Research Council, University of Pretoria, Pretoria, South Africa; 3 PathCare Vermaak Histopathology Laboratory, Pretoria, South Africa; 4 Pretoria Oral Health Care Centre, Corner of Steve Biko and Dr Savage Roads, Pretoria, 0084 South Africa

**Keywords:** Odontogenic neoplasms, Ameloblastoma, Ameloblastic carcinoma, Immunohistochemistry, DNA ploidy, Flow cytometry

## Abstract

**Purpose:**

This study aimed to investigate the role of DNA ploidy and proliferation index in distinguishing ameloblastoma (AB) from ameloblastic carcinoma (AC).

**Methods:**

The study included 29 ACs, 6 conventional ABs that transformed into ACs, and a control cohort of 20 conventional ABs. The demographics and clinicopathologic details of the included cases were summarised and compared. The Ki-67 proliferation index was scored using the QuPath open-source software platform. DNA ploidy analysis was performed using high-resolution flow cytometry.

**Results:**

The cohort of ABs presented at an overall younger age compared to both primary and secondary ACs. There was a statistically significant difference between the median duration of the tumour when comparing primary and secondary ACs, with ACs presenting with longer durations than the AB cohort. All cases of AC showed a relatively high median proliferation index of 41.7%, with statistically significant higher scores compared to ABs. DNA ploidy analysis showed that all cases in the AB cohort were diploid. Two diploid cases of AB that transformed into ACs were aneuploid when the corresponding secondary AC was analysed. Fourteen cases of AC were diploid and 12 were aneuploid, with no statistically significant association found between DNA ploidy status of primary and secondary ACs. A statistically significant difference was noted when the DNA ploidy status of ABs was compared to that of ACs. When comparing the Ki-67 proliferation score of ACs to their DNA ploidy status, no statistically significant association was noted.

**Conclusion:**

DNA ploidy analysis and proliferation index via Ki-67 IHC are useful ancillary tests that may be used to support a diagnosis of AC and may assist in distinguishing between challenging cases of AB and AC.

## Introduction

Odontogenic lesions encompass a group of entities, broadly classified into cysts and tumours, derived from remnants of the tooth germ [1, 2]. The odontogenic tumours are further subclassified based on their constituent odontogenic tissue, being purely epithelial, purely mesenchymal or mixed [2, 3]. Almost all odontogenic tumours are considered neoplastic and subdivided into benign and malignant entities [3]. The majority of odontogenic tumours fall into the benign category, whereas malignant odontogenic entities are significantly rarer [1, 2]. In general, literature is particularly sparse on malignant odontogenic neoplasms, with a large proportion of the current knowledge derived from case reports or small case series [4].

The latest 5th Edition of the WHO classification includes ameloblastic carcinoma (AC) within the category of malignant odontogenic tumours, simply defining AC as a primary odontogenic carcinoma histologically resembling ameloblastoma (AB). AC is most common in this group of odontogenic malignancies, constituting approximately 30% of all cases in this category [3]. ACs are further subdivided into primary cases, arising de novo, and secondary cases, arising in a preexisting AB [1, 3, 5–7].

ACs have a vague diagnostic threshold, making their diagnosis challenging [1, 2], particularly in cases with intermediate histopathologic features between benign and malignant odontogenic neoplasms. Previous studies have found differences in the clinico-radiologic presentation of ABs and ACs, which may aid in their diagnosis. Clinically, ACs presented with higher rates of pain and paraesthesia compared to ABs. Radiologically, poorly demarcated borders and cortical destruction were more common in ACs versus ABs [8–10]. Authors have also proposed various ancillary studies, including determining the proliferation index of a tumour via immunohistochemistry (IHC), to assist in diagnosing challenging cases [11]. The proliferation index of a tumour is defined as the percentage of cells in the tumour actively undergoing cell division. This gives insight into a tumour’s growth rate, aggressiveness and biological behaviour. Ki-67 is a protein expressed by proliferating cells in the various stages of the cell cycle, except for the resting phase, and has been used as a diagnostic tool to differentiate entities with similar histopathologic appearances, including benign versus malignant odontogenic tumours [4, 9, 11–13].

Carcinogenesis is frequently associated with genomic changes, chromosomal alterations, DNA content changes and variations in the cell cycle of neoplastic cells [14, 15]. Hedley et al. first developed a method for obtaining cell suspensions from paraffin-embedded Sect. [16]. This method culminated in the ability to determine DNA content abnormalities from routine formalin-fixed paraffin-embedded (FFPE) tissue blocks [17]. DNA ploidy analysis by flow or image cytometry allows for measuring DNA content, termed ploidy, in tumour cell populations by comparing the integrated optical density of the nuclei of interest with control nuclei [18, 19]. In the context of oral and maxillofacial pathology, Grassel-Pietrusky et al. first used flow cytometry (FC) to examine leukoplakias with varying degrees of epithelial dysplasia [20]. Since then, it has been used to predict the prognostic implications of potentially malignant and malignant epithelial lesions in the oral cavity and malignant salivary gland tumours [14, 17, 18, 21–23]. DNA ploidy analysis has also been postulated by several histopathologists to assist in distinguishing between benign and malignant odontogenic entities. However, to date, there have been limited studies in the literature analysing DNA ploidy status of odontogenic lesions [4, 24–28].

The current study aims to investigate the use of DNA ploidy analysis and proliferation index determined via Ki-67 IHC in distinguishing AB from AC. In contrast to previous studies, this study utilises a high-resolution flow cytometer with a UV light source to measure DNA content. The UV light source allows for the use of a DNA-specific dye, 4′,6-diamidino-2-phenylindole (DAPI), instead of the more widely used propidium iodide dye. DAPI enhances binding to DNA by 200-fold, allowing for more accurate interpretation [29, 30]. Additionally, utilising high-resolution FC allows for the use of FFPE tissue over fresh tissue without lowering the sensitivity of the measurements [29, 30].

The findings of this study will hopefully serve as an adjunct to the essential histopathologic diagnostic criteria, ultimately aiding the diagnosis of these rare and often diagnostically challenging odontogenic malignancies.

## Materials and Methods

### Case Selection

Ameloblastic carcinomas diagnosed within the Department of Oral and Maxillofacial Pathology at the University of Pretoria between 2002 and 2022 (20-year period) were retrieved from the histopathologic database. The patient demographics, clinical features and available radiologic imaging were recorded and ultimately assessed to assist the diagnostic process. The principal investigator (LR) and an experienced Oral and Maxillofacial Pathologist (WvH) reviewed all cases to confirm the diagnosis according to the latest WHO Classification diagnostic criteria. These include a histopathologic resemblance to ameloblastoma and evidence of cytologic atypia [3].

The original haematoxylin and eosin (H&E)-stained slides and the formalin-fixed paraffin-embedded (FFPE) tissue blocks were then accessed. The best representative FFPE tissue block, with adequate tumoural tissue and without excessive necrosis, was selected to perform additional studies. These FFPE tissue blocks were optimally processed and were stored at temperatures maintained in the range of 17–22 °C and protected from direct light to maintain tissue integrity. Decalcified tissue or tissue that contained osseous material were not used for further ancillary testing.

After confirmation of the diagnosis of AC, the histopathologic database was re-assessed to subclassify the case as either a primary or a secondary AC. Records of a previous diagnosis of ameloblastoma were required for secondary cases of ameloblastic carcinoma. If available, the previous diagnosis of ameloblastoma was re-assessed and confirmed, and the FFPE tissue blocks accessed.

A control cohort of 20 conventional ameloblastomas was also selected from the same histopathologic database of the Department within the same study period. These cases encompassed the histopathologic spectrum of conventional ameloblastomas and included both mandibular and maxillary cases. All cases were also re-assessed by the same oral and maxillofacial pathologists to confirm the diagnosis as stipulated by the WHO Classification [3].

### Immunohistochemistry

Immunohistochemical analysis for Ki-67 (Dako, RTU, clone: MIB-1, Agilent Technologies, Singapore) was performed on freshly cut 4 μm sections from the representative FFPE tissue block from included AB and AC cases. Staining was performed on a Ventana Benchmark GX automated system (Ventana Medical Systems Inc, Tucson, Arizona, USA). Standard antigen retrieval was performed using Ventana CC1 cell conditioning solution for 32 min, followed by incubation with the primary antibody for 38 min, and detection using the Ventana OptiView DAB detection kit. The sections were then counterstained with haematoxylin, dehydrated in graded alcohol solutions, cleared in xylene and mounted with DPX mounting media.

**Fig. 1:**
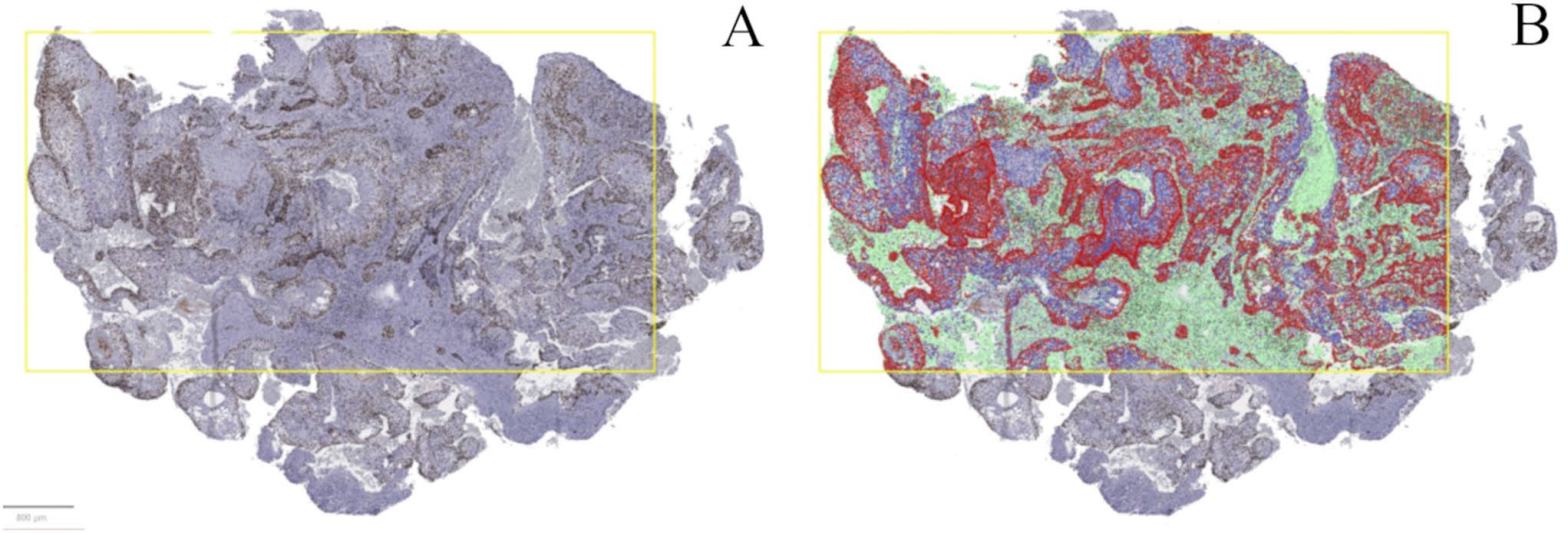
QuPath methodology for whole slide image analysis. (**A**) Selection of a rectangular region of interest in a hotspot area on the digitally scanned slide. (**B**) Training the object classifier to distinguish between stroma (green) and the tumour to determine the percentage of positively stained tumour cells alone. Positive nuclear cell detection was run within the tumour cells, with positive cells marked red and negative cells marked purple

The Ki-67 proliferation index was determined in hotspot regions without significant inflammation using AI software (QuPath open-source software platform, Version 0.5.1), similar to the methodology employed in a recent article by Robinson et al. [9] (Fig. 1).

### DNA Ploidy

DNA ploidy analysis via high-resolution FC was performed on sections from the same FFPE tissue block used for IHC analysis. Sections were prepared according to the modified Hedley method [31]. Four to six 40-µm sections from each case were cut, wrapped in 50-µm nylon mesh, placed in a histocassette and manually dewaxed and hydrated in distilled water overnight. These sections underwent optimal digestion in subtilisin Carlsberg solution at 37 °C for 120 min. The cell suspension was then stained with DAPI (Research Organics, Cleveland, Ohio, USA). As far as possible, a constant dye-to-DNA substrate ratio was maintained. High-resolution FC was carried out using a CyFlow Space flow cytometer equipped with a 488 nm laser (Partec, Münster, Germany). Before performing DNA ploidy analysis, the flow cytometer underwent adequate calibration using DNA control UV (Partec, Münster, Germany). Good quality, representative DNA histograms were then plotted and assessed. Based on published consensus guidelines [32], the DNA content was counted, whereby the minimum number of cells or nuclei required to reproducibly detect DNA content abnormalities is ~ 10,000. The coefficient of variance (CV), which reflects the sensitivity of flow cytometric DNA measurements, were calculated and recorded in each case, according to the recommended guidelines [32]. Stromal and inflammatory cells, present in all samples, provided the internal diploid standard to allow the identification of DNA aneuploidy. The DNA index (DI) was calculated for each case relative to internal diploid controls (lymphocytes; DI = 1.0). The DI is expressed by the quotient of the respective modal peak values and reflects the discrepancy between the aneuploid DNA content and the internal diploid DNA amount [15].

A graphic interpretation of the histograms was performed, whereby the following principles were used in determining DNA ploidy [19, 32]:

I. A tumour with a single G_0_/G_1_ peak with a DI of 1.0 was regarded as being diploid;
II. Any evidence of an additional G_0_/G_1_ peak (< or > 1) indicated the presence of an aneuploid stemline. DNA tetraploidy was recorded as a distinct category of DNA aneuploidy, due to previous reports of prognostic significance in some tumour types [33]. An aneuploid cell population with tetraploid DNA content was noted when the G_2_M peak was present in the ‘octaploid channel’ with more cell content than in the ‘hexaploid channel’, excluding the possibility of clump formation.

Two authors (LR and WvH) interpreted all flow cytometric histograms without knowledge of the clinical history and the histopathologic diagnosis.

DNA ploidy analysis was performed on the following cases:

- Ameloblastic carcinomas (primary and secondary);
- Ameloblastomas that transformed into ameloblastic carcinomas;
- A cohort of conventional ameloblastomas.

### Statistical Analysis

The clinical features, immunohistochemical, and DNA ploidy results were recorded using Microsoft Excel (Version 2019), and subsequent statistical analysis of the data was performed using SPSS software 29.0 (IBM Corporation, New York, NY, USA). The data consisted of categorical data (sex, DNA ploidy status) and continuous data (age, duration, Ki-67 proliferation scores). The distribution of continuous data was confirmed using the Shapiro-Wilk test. Descriptive statistics were reported using means with standard deviations for normally distributed data and median and interquartile ranges for non-normally distributed data. Categorical data was recorded using frequencies and percentages. Correlations between categorical data were assessed using Chi-squared tests. Fisher’s exact test was used as a reasonability check when the underlying assumptions of Chi-squared tests were violated. When comparing continuous data between two categorical variables, Mann-Whitney U tests and unpaired t-tests were used depending on the normality of their distributions. Throughout the statistical analysis, correlations with a two-sided asymptotic significance (*P*-value) of less than 0.05 were considered statistically significant.

## Results

**Fig. 2:**
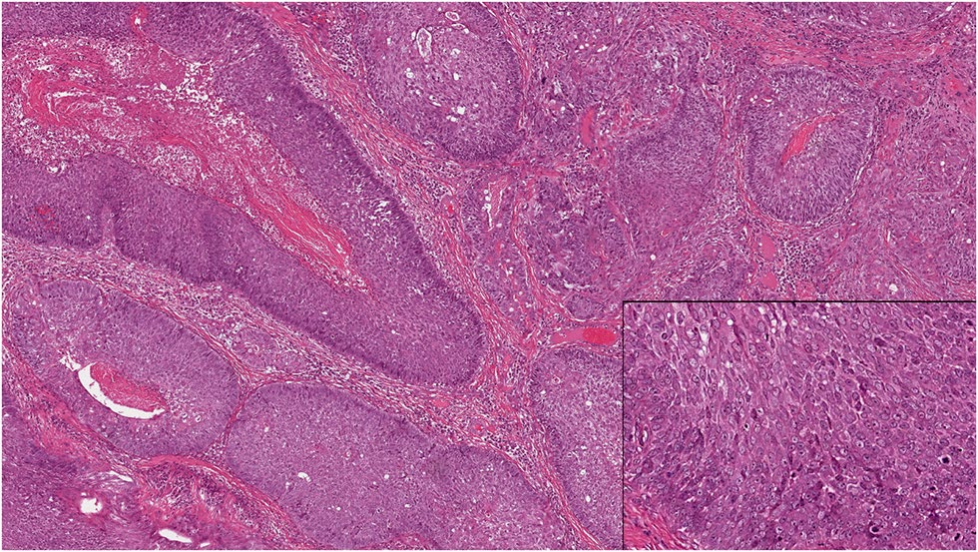
Histopathologic features of ameloblastic carcinomas. Essential diagnostic criteria, including resemblance to ameloblastoma (original magnification x 40). Insert: Evidence of high-grade cytologic atypia (original magnification x 100)

During the study period, 29 cases of AC were diagnosed at the institution, with 17 classified as primary AC and 12 as secondary AC arising from a previous AB. All cases of AC met the essential diagnostic criteria according to the latest WHO Classification (Fig. 2) [3]. The FFPE tissue blocks were available for six conventional ABs that transformed into ACs.

Table 1 summarises and compares the clinical features and DNA ploidy results of ABs and ACs.

**Table 1:** Clinical features and DNA ploidy results of ameloblastomas and ameloblastic carcinomas

	Ameloblastomas (ABs)							Ameloblastic carcinomas (ACs)							ABs vs. ACs
	ABs		Transformed ABs		*P*-value	Total ABs		Secondary ACs		Primary ACs		*P*-value	Total ACs		*P*- value
	*n* = 20	%	*n* = 6	%		*n* = 26	**%**	*n* = 12	%	*n* = 17	%		*n* = 29	**%**	
**Clinical features**															
Age (years)—mean, range, (standard deviation)	35.4	10–94 (19.4)	45	33–62 (11.2)	*0.149*	**37.58**	**10–94 (18.3)**	42.2	(10.5)	43.8	(19.7)	*0.801*	**43.1**	**4.0–76.0 (16.5)**	*0.244*
Sex (M: F)	11:9	1.2:1	6:0		*0.063*	**17:9**	**(1.9:1)**	8:4	2:1	12:5	2:1	*0.568*	**20:9**	**2:1**	*1.000*
Clinical duration of the lesion (months)—median, interquartile range	24^a^	(6–48)	13.5	(8.25-43)	*0.898*	**24**	**(1-180)**	54	(31.5-116.5)	6	(3–12)	0.006*	**40** ^c^	**(4.5–60)**	*0.847*
**DNA ploidy**															
*Diploid*	19^b^	100%	5	83.3%	*0.157*	**24**	**96%**	8	66.7%	6^d^	42.9%	*0.144*	**14**	**52%**	0.002*
*Aneuploid*	0	0%	1	16.7%		**1**	**4%**	4	33.3%	8^d^	57.1%		**12**	**48%**	

^a^ Duration was not reported in one case

^b^ Ploidy failed in one case

^c^ Duration was not reported in 12 cases

^d^ Ploidy failed in three cases

*Statistically significant

In the current study, the mean age of patients diagnosed with AC was approximately 43 years, similar for both primary and secondary cases. In contrast, although not statistically significant, ABs presented at an overall younger age. The male-to-female ratio for both primary and secondary ACs showed a male predominance, whereas, within the cohort of ABs, an almost equal sex ratio was noted. Although only a small sample, all six cases of AB that transformed into AC were seen in male patients. The median duration of the tumour, reported by the patient, for primary and secondary ACs were markedly different at 6 and 54 months, respectively, a statistically significant finding. Overall, the clinical median duration of the tumour was longer for all AC cases compared to the AB cohort.

### Proliferation Index

**Fig. 3:**
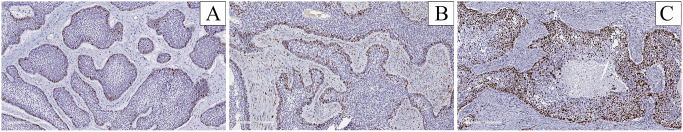
Representative Ki-67 IHC for (**A**) AB, (**B**) AB that transformed into AC, and (**C**) AC (original magnifications x 40)

Regarding the proliferation index via Ki-67 IHC, all AC cases showed a statistically significant higher (*p* < 0.001) median proliferation index (41.7%, interquartile range: 29.1–59.9%) compared to AB cases (9%, interquartile range: 2-12.5%) [9]. The six ABs that transformed into ACs had a median proliferation index of 10% (interquartile range: 5–20%) [9]. The staining pattern of Ki-67 IHC was mainly confined to the basal ameloblast-like cells in AB cases. In contrast, AC cases showed an expanded Ki-67 staining pattern due to the more solid, compact sheets of basaloid epithelium with a loss of the stellate reticulum-like cells, often seen in cases of AC (Fig. 3) [3].

## DNA Ploidy

Of the total cases analysed via high-resolution FC, four cases (3 ACs and a single AB) failed and could not be interpreted. These four cases did not yield sufficient viable cells for evaluation. This was likely due to pre-analytical factors such as tissue fixation and processing. Overall, a mean of 52,027 cells were counted, and the mean coefficient of variance was 3.5% (standard deviation = 1.27).

**Fig. 4:**
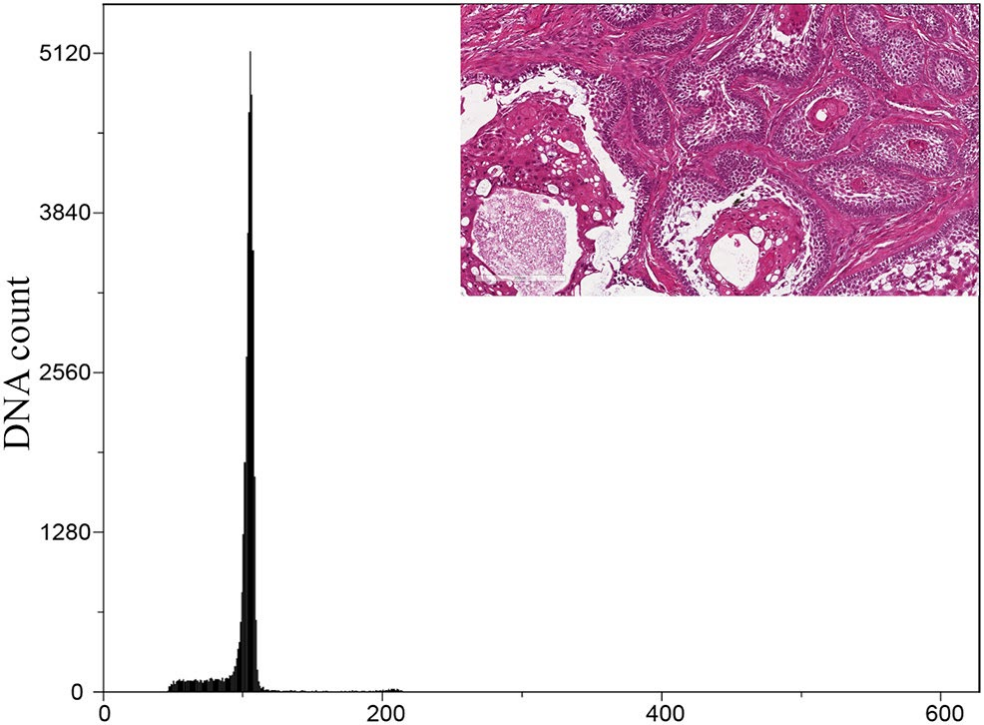
Representative DNA histogram of a diploid AB case. Insert: H&E-stained section of corresponding AB case (original magnification x 40)

**Fig. 5:**
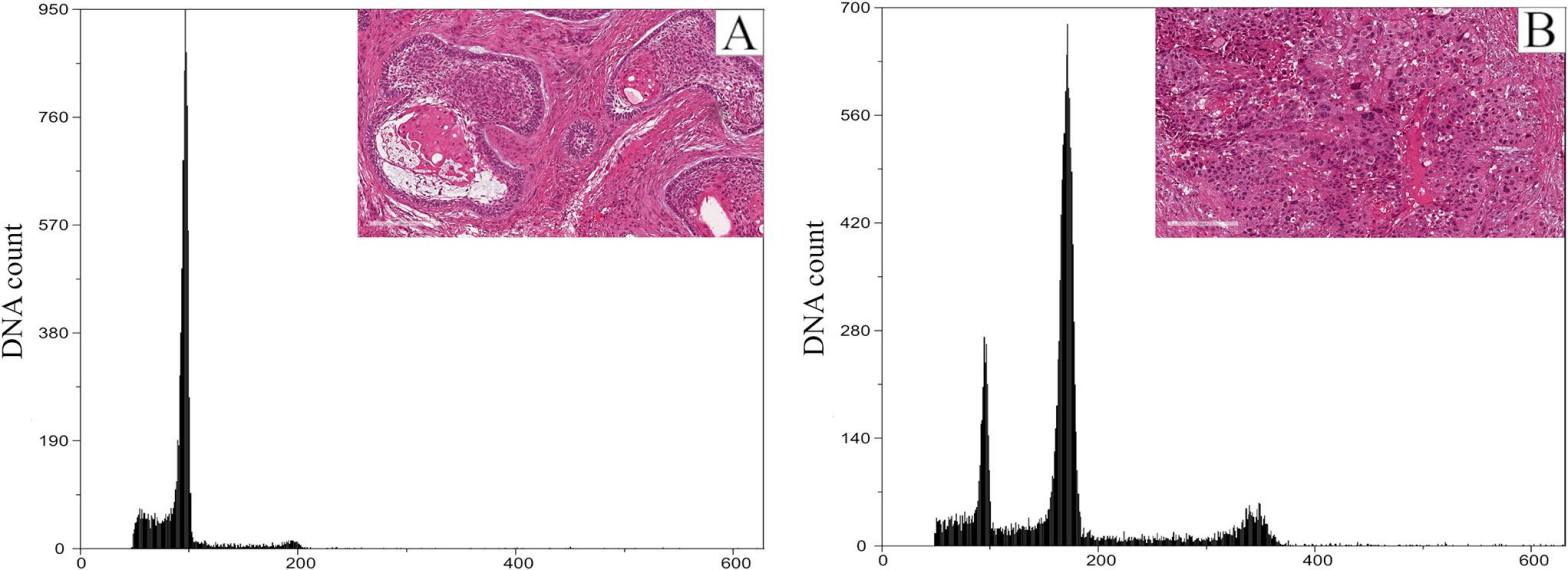
Representative DNA histograms of (**A**) diploid AB and (**B**) the corresponding secondary AC which was aneuploid. Inserts: H&E-stained sections of (**A**) corresponding AB and (**B**) secondary AC (original magnifications x 40)

**Fig. 6:**
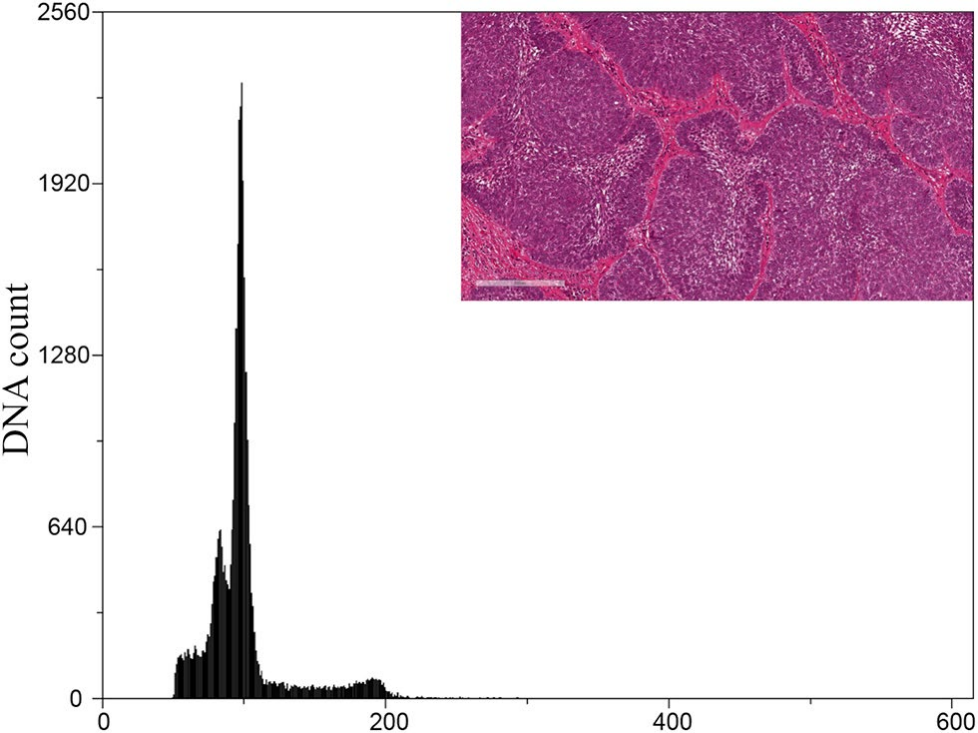
Representative DNA histogram of an aneuploid AC case. Insert: H&E-stained section of corresponding AC case (original magnification x 40)

Within the cohort of ABs, all cases were diploid (Fig. 4). Only one case of the six ABs that transformed into ACs was aneuploid, of which the corresponding AC was also aneuploid. Two diploid cases of AB that transformed into AC were aneuploid when the corresponding secondary AC was analysed (Fig. 5). Of the total ACs, 14 were diploid, and 12 were aneuploid (Fig. 6). Only a single case of AC showed an aneuploid cell population with tetraploid DNA content. There was no statistical significance between primary and secondary ACs regarding DNA ploidy status. However, a statistical significance was noted when the DNA ploidy status of ABs was compared to that of ACs.

### Proliferation Index Versus DNA Ploidy

No statistically significant associations were noted when comparing the Ki-67 proliferation score of ACs to the DNA ploidy status (*p* = 0.206).

## Discussion

Ameloblastic carcinomas are rare entities that may pose diagnostic dilemmas [1, 3], particularly in distinguishing cases with intermediate histopathologic features. This difficulty is further exacerbated by poorly defined diagnostic thresholds [7]. Further studies are required to better elucidate this entity and improve the diagnostic work-up of potential cases.

The current study of 29 cases of AC represents a sizeable single-centre study, albeit in the same population group. In this study, the mean age of presentation was 43 years, lower than a recent systematic review [8]. Interestingly, the mean age of primary and secondary ACs was similar. The current study also included a control cohort of ABs, which presented at a comparatively younger age, but was similar to a large single-centre study on ABs within the same population group [10]. ACs showed a male predominance in the current study, contradicting the reported literature [8]. Of note, in the current study, the sample of ABs that transformed into ACs only presented in male patients. Due to the relatively small sample, this finding should be viewed cautiously. As expected, the median clinical duration of tumours in the current study was markedly higher for secondary AC cases than for primary cases.

Determining the proliferative index of a tumour using Ki-67 IHC has been used as an ancillary tool to differentiate entities with similar histopathologic appearances [4, 11–13]. The limitation of Ki-67 IHC revolves around its interpretation and the subjective nature thereof. A recent systematic review recorded a vast range of Ki-67 scores, between 5 and 80% [8]. Regardless of this limitation, literature constantly quotes higher mean proliferation indices in ACs compared to ABs [6, 34]. The findings of the current study mirrored the literature, finding a statistically significant higher Ki-67 score in cases of AC compared to ABs included in the study [9]. A noteworthy difference in the current study stemmed from using an automated proliferation index counter, thereby increasing the number of cells analysed and reducing the subjective nature and reported range in interpreting the IHC stain.

Tumour neoplastic cells, in particular malignancies, are known to contain DNA content changes and cell cycle variations [14]. Utilising this principle, cytometric measurements of nuclear DNA content by FC have helped distinguish benign and malignant neoplasms [25, 26, 35]. Additionally, measuring DNA by FC may serve as a valuable adjunct to histopathologic evaluation when grading tumours [23, 26]. The nuclear content of several carcinomas, including ovarian, breast, thyroid, and oral, has also provided important prognostic information [36–39].

Flow cytometry is an accurate, rapid technique to determine the DNA ploidy of individual cells in a suspension [15, 22, 40]. The nuclei are stained with a fluorescent dye and sent through a flow channel, where the dye is excited by a laser beam or mercury lamp. DNA content is measured based on the proportional binding of the DNA-specific dye to nuclear DNA, resulting in a DNA content histogram of the entire cell population [40, 41]. The current study utilised high-resolution FC, whereby DAPI was used instead of the dye propidium iodide. This, linked to the UV light source, produces high-quality histograms [41], as reflected in the current study, which, despite using FFPE tissue, produced an excellent mean coefficient of variance.

Several previous studies have successfully used FC on oral squamous cell carcinomas (SCCs) to correlate DNA ploidy status with tumour grading and staging, tumour progression, recurrence rates and metastatic potential [15, 17, 22, 29, 42, 43]. Salivary gland tumours (SGTs) have also undergone DNA ploidy analysis to distinguish benign versus malignant entities and to correlate with the histopathologic grading of malignant SGTs [14, 21, 23]. Concerning odontogenic lesions, a case report by High et al., utilised FC to determine DNA aneuploidy in an odontogenic keratocyst that underwent malignant transformation. In this case report, the subsequent carcinoma was also aneuploid, both locally and in the metastatic tumour deposit [44]. An early study by Muller et al. utilised image and FC to analyse DNA content in primary and recurrent ABs and AC cases [25]. They analysed 17 primary and five recurrent ABs and five ACs, with fourteen (82%) primary ABs and three (60%) of the recurrent cases being diploid. They found no statistically significant difference in ploidy analysis between primary and recurrent ABs or among the different histological variants included in the study. Importantly four (80%) of the five ACs were aneuploid. FC was performed on three AC cases with 100% concordance between the image and flow cytometric data [25]. The authors concluded that aneuploidy in this tumour group strongly indicates malignancy [25]. This contrasts with the study by Mahmoud et al., in which all eight cases of AC were diploid [27]. More recently, Penafort et al., found that DNA ploidy may act as an ancillary tool in distinguishing AB and AC [28]. Unfortunately, this study included a relatively small sample of five ACs, and DNA ploidy analysis was conducted using conventional FC with propidium iodide dye.

In the current study, almost all ABs within the cohort were diploid, with only a single case of AB that transformed into AC interpreted as aneuploid. Notably, the corresponding AC was also aneuploid. Many authors believe that aneuploidy is a cause rather than the result of malignant transformation, forming an essential part of carcinogenesis. This implies that aneuploidy in tumours at an early stage suggests that abnormal DNA content may be associated with tumour progression [18, 40, 42, 45]. Further studies are required in this regard. Interestingly, in the current study, two cases of AB that transformed into ACs were reported as diploid, with their subsequent corresponding secondary AC being aneuploid. This implies that DNA content changes occurred as part of the malignant transformation. Similar studies on oral SCCs found that aneuploidy formation represents a late but ultimate event in the disease process and contributes to the behaviour of the carcinoma [15, 17, 43]. Another study focusing on genito-urinary neoplasms also found a decrease in the proportion of diploid cases with tumour progression [33].

Regarding primary and secondary ACs, no statistically significant difference was noted between the subtypes of AC and DNA ploidy status, a finding not yet reported in the literature. A significant association was noted when comparing the DNA ploidy status of ABs to ACs. Although not all cases of AC were aneuploid, this affirms the notion by Muller et al. that aneuploidy supports, but is not diagnostic, of malignancy [25]. Tetraploid cells are genomically unstable and known to facilitate tumorigenesis, arising from diploid progenitors by endomitosis and subsequent loss of chromosomes [42, 46, 47]. Additionally, tumour tetraploidy may play a role in tumour progression and relapse following chemotherapeutic treatment [46, 47]. In the current study, only a single case of primary AC showed an aneuploid cell population with tetraploid DNA content, limiting further correlations. Finally, no correlation was noted between DNA ploidy status and Ki-67 proliferation scores for cases of AC in the current study, implying that the two variables are independent features in diagnosing ACs.

A possible pitfall of DNA ploidy analysis by FC is tumour heterogeneity reported in oral SCCs, SGTs and ACs [21, 25, 48, 49]. To counter this drawback, researchers often advocate the repetition of ploidy analysis to exclude spurious diploid cases [21]. Additionally, maintaining excellent CV values, as low as possible, which are reflected on the graph as narrow peaks. This is critical to correctly diagnose diploid cases without missing peridiploid aneuploid peaks, which cannot be detected with high CV values [15]. The mean CV value in the current study was 3.5%, while most studies on oral SCCs using archival material described values higher than 5% when using propidium iodide as a DNA marker [50, 51]. Other studies on DNA FC in odontogenic carcinomas did not reveal the CV values [25–28], apart from the case report by High et al., which quoted a CV of 7.75% [44]. The use of high-resolution FC in the current study contributed to the excellent reported CV values, despite using FFPE material [29, 30].

The limitations of the current study included the small sample size from a single centre and limited information from clinical records due to the retrospective nature of the study. ACs are rare, highlighting the importance of future collaborative studies to pool all available data for further analyses. Possible case selection bias was mitigated by two oral and maxillofacial pathologists with extensive experience in odontogenic pathology, who confirmed the diagnosis of each case according to the latest diagnostic criteria [3].

## Conclusion

Due to statistically significant differences in Ki-67 IHC and DNA ploidy status between ABs and ACs, these ancillary tests serve as valuable adjuncts to assist in distinguishing challenging cases. High proliferation indices and DNA aneuploidy are useful findings to support a diagnosis of AC. Additional large series studies, utilising molecular diagnostic techniques, are required to support the findings of the current study and to expand on the current understanding of this rare malignant odontogenic tumour.

## Data Availability

No datasets were generated or analysed during the current study.
